# Supplementary material for: Preliminary Comparisons of Tender Shoots and Young Leaves of 12 Mulberry Varieties as Vegetables and Constituents Relevant for Their Potential Use as Functional Food for Blood Sugar Control
Source: Plants (Basel). 2023 Nov 2;12(21):3748. doi: 10.3390/plants12213748 (PMC10650630; doi:10.3390/plants12213748)
Supplement: Supplementary file 1 [file plants-12-03748-s001.zip › Table S1 The content of main carbohydrates and SP in tender shoots and leaves of differential mulberry varieties.pdf]

Table S1. The content of main carbohydrates and SP in tender shoots and leaves of differential mulberry varieties.

	SP (mg/g DW)		Sucrose (mg/g DW)		Glucose (mg/g DW)		Fructose (mg/g DW)	
	Tender shoots	Leaves	Tender shoots	Leaves	Tender shoots	Leaves	Tender shoots	Leaves
VM1	53.13±5.50 ^{bc}	21.35±0.23 ^{b}	9.09±0.42 ^{d}	55.14±0.57 ^{d}	4.76±0.11 ^{b}	21.35±0.23 ^{b}	12.91±0.73 ^{b}	12.71±0.69 ^{a}
VM5	50.91±0.11 ^{d}	2.35±0.09 ^{j}	15.26±1.12 ^{a}	39.29±1.20 ^{h}	1.54±0.09 ^{h}	2.35±0.09 ^{j}	5.38±0.45 ^{g}	6.20±0.41 ^{f}
VM7	55.57±0.07 ^{b}	4.89±0.14 ^{i}	7.67±0.28 ^{e}	42.56±2.00 ^{gh}	1.06±0.06 ^{i}	4.89±0.14 ^{i}	6.17±0.82 ^{f}	4.94±0.41 ^{h}
VM9	54.27±0.10 ^{b}	6.53±0.07 ^{g}	4.58±0.16 ^{f}	52.74±1.12 ^{d}	1.37±0.07 ^{h}	6.53±0.07 ^{g}	10.32±0.66 ^{d}	7.31±0.43 ^{e}
VM10	56.41±0.17 ^{b}	7.73±0.16 ^{f}	2.23±0.07 ^{h}	45.00±2.38 ^{fg}	2.37±0.09 ^{g}	7.73±0.16 ^{f}	7.87±0.36 ^{e}	9.18±0.45 ^{cd}
VM12	52.36±0.28 ^{c}	15.76±0.61 ^{c}	12.86±0.86 ^{b}	64.40±2.76 ^{c}	7.61±0.14 ^{a}	15.76±0.61 ^{c}	15.27±0.85 ^{a}	8.70±0.47 ^{d}
VM13	58.39±0.19 ^{a}	5.38±0.13 ^{h}	3.51±0.31 ^{g}	47.60±2.15 ^{ef}	3.49±0.21 ^{d}	5.38±0.13 ^{h}	11.72±0.92 ^{c}	6.92±0.35 ^{ef}
VM16	50.68±0.11 ^{d}	11.02±0.28 ^{e}	2.28±0.15 ^{h}	46.39±1.34 ^{ef}	2.38±0.20 ^{g}	11.02±0.28 ^{e}	9.71±0.27 ^{d}	11.66±0.80 ^{b}
VM18	45.87±0.20 ^{e}	31.15±1.71 ^{a}	4.17±0.35 ^{fg}	77.24±2.72 ^{a}	4.09±0.19 ^{c}	31.15±1.71 ^{a}	13.01±0.69 ^{b}	11.95±0.62 ^{ab}
VM19	54.88±0.21 ^{b}	22.45±0.82 ^{b}	3.43±0.09 ^{g}	49.09±1.35 ^{e}	2.76±0.12 ^{ef}	22.45±0.85 ^{b}	13.00±0.55 ^{b}	11.41±0.77 ^{b}
VM22	52.21±0.32 ^{c}	13.72±0.54 ^{d}	3.87±0.21 ^{fg}	70.17±3.12 ^{b}	2.98±0.13 ^{e}	13.72±0.54 ^{d}	8.19±0.53 ^{e}	9.84±0.31 ^{c}
VM23	54.35±0.17 ^{b}	4.75±0.04 ^{i}	10.21±0.64 ^{c}	61.68±1.78 ^{c}	2.67±0.07 ^{f}	4.75±2.04 ^{i}	8.00±0.33 ^{e}	5.83±0.31 ^{g}
Mean	53.25	12.26	6.60	54.27	3.09	12.26	10.13	8.89
SD	3.24	2.50	4.36	11.76	1.79	8.89	3.09	2.66
CV	6.07	20.39	66.14	21.67	58.00	72.51	30.52	29.89

The different small letter superscripts within the same column represent significant differences ($p < 0.05$) (ANOVA and LSD test). The data are mean values of three replicates and standard deviation of the mean.
