# Supplementary material for: Preliminary Comparisons of Tender Shoots and Young Leaves of 12 Mulberry Varieties as Vegetables and Constituents Relevant for Their Potential Use as Functional Food for Blood Sugar Control
Source: Plants (Basel). 2023 Nov 2;12(21):3748. doi: 10.3390/plants12213748 (PMC10650630; doi:10.3390/plants12213748)
Supplement: Supplementary file 1 [file plants-12-03748-s001.zip › Table S2 DNJ and Vc VC content in tender shoots and leaves of differential mulberry varieties.pdf]

**Table S2.** DNJ and VC content in tender shoots and leaves of differential mulberry varieties.

| Varieties | VC (mg/100g DW) | | DNJ（mg/g DW） | |
|---|---|---|---|---|
| | Tender shoots | Leaves | Tender shoots | Leaves |
| *VM1* | 153.52±12.53 [a] | 128.72±14.98 [ab] | 4.74±0.48 [e] | 2.08±0.16 [g] |
| *VM5* | 136.83±11.27 [ab] | 120.45±10.55 [ab] | 7.23±0.95 [d] | 5.06±0.23 [c] |
| *VM7* | 138.39±14.40 [ab] | 126.38±11.04 [ab] | 7.33±0.64 [d] | 9.56±0.94 [a] |
| *VM9* | 151.96±15.86 [ab] | 126.07±11.00 [ab] | 7.96±0.42 [b] | 2.93±0.60 [e] |
| *VM10* | 137.14±10.89 [ab] | 133.87±5.64 [ab] | 4.92±0.29 [e] | 4.86±0.84 [c] |
| *VM12* | 141.67±13.58 [ab] | 118.58±11.13 [b] | 7.89±0.15 [bc] | 3.64±0.57 [d] |
| *VM13* | 134.18±16.43 [b] | 122.01±4.93 [ab] | 8.92±1.01 [a] | 9.84±0.73 [a] |
| *VM16* | 144.48±11.69 [ab] | 136.99±12.98 [a] | 6.24±0.84 [d] | 2.450±0.57 [f] |
| *VM18* | 129.97±5.12 [b] | 133.24±8.59 [ab] | 8.06±0.90 [b] | 3.58±0.47 [d] |
| *VM19* | 153.68±13.24 [a] | 125.76±9.65 [ab] | 8.29±0.65 [b] | 8.83±0.54 [b] |
| *VM22* | 149.47±17.89 [ab] | 117.80±12.68 [b] | 7.36±0.70 [cd] | 8.84±0.72 [b] |
| *VM23* | 143.07±10.90 [ab] | 125.76±18.33 [ab] | 6.93±0.91 [d] | 2.85±0.12 [e] |
| Mean | 141.11 | 126.75 | 7.16 | 5.38 |
| SD | 7.93 | 6.11 | 1.29 | 3.01 |
| CV | 5.55 | 4.84 | 17.98 | 55.92 |

The different small letter superscripts within the same column represent significant differences ($p < 0.05$) (ANOVA and LSD test). The data are mean values of three replicates and standard deviation of the mean.
