# Supplementary material for: Preliminary Comparisons of Tender Shoots and Young Leaves of 12 Mulberry Varieties as Vegetables and Constituents Relevant for Their Potential Use as Functional Food for Blood Sugar Control
Source: Plants (Basel). 2023 Nov 2;12(21):3748. doi: 10.3390/plants12213748 (PMC10650630; doi:10.3390/plants12213748)
Supplement: Supplementary file 1 [file plants-12-03748-s001.zip › Table S3 Total antioxidant activities of tender shoots and leaves in differential mulberry varieties.pdf]

Table S3. Total antioxidant activities of tender shoots and leaves in differential mulberry varieties.

Varieties	ABTS (μmol TE/g DW)		DPPH (μmol TE/g DW)		FRAP (μmol TE/g DW)	
	Tender shoots	Leaves	Tender shoots	Leaves	Tender shoots	Leaves
VM1	139.68±16.62 ^{b}	130.17±12.8 7^{bc}	52.48±12.5 7^{de}	37.50±5.49 ^{d}	125.81±11.87 ^{e}	127.50±10.53 ^{c}
VM5	153.00±15.72 ^{ab}	117.09±11.53 ^{cd}	64.81±7.27 ^{abcd}	61.75±4.14 ^{ab}	134.81±12.53 ^{e}	128.42±8.65 ^{c}
VM7	186.35±20.04 ^{a}	136.11±16.76 ^{bc}	72.72±7.41 ^{ab}	68.11±10.63 ^{a}	169.38±5.60 ^{a}	144.78±14.73 ^{abc}
VM9	158.71±11.15 ^{b}	139.92±13.36 ^{abc}	59.20±4.08 ^{bcde}	53.25±1.92 ^{bc}	135.87±6.39 ^{e}	133.25±15.88 ^{bc}
VM10	154.66±14.18 ^{b}	142.06±14.82 ^{ab}	63.85±8.04 ^{abcd}	40.72±4.44 ^{d}	137.19±7.58 ^{de}	134.06±7.65 ^{bc}
VM12	141.58±23.64 ^{b}	143.72±16.42 ^{ab}	47.74±8.42 ^{e}	43.72±4.13 ^{cd}	137.74±17.55 ^{de}	123.72±4.13 ^{c}
VM13	159.89±15.10 ^{ab}	111.61±6.29 ^{d}	72.38±9.52 ^{ab}	64.95±6.62 ^{a}	157.71±18.50 ^{abcd}	154.95±21.02 ^{ab}
VM16	136.60±11.64 ^{b}	161.59±17.69 ^{a}	56.85±8.05 ^{cde}	61.59±8.83 ^{ab}	143.52±5.27 ^{bcde}	141.59±17.18 ^{abc}
VM18	158.94±14.85 ^{ab}	144.67±13.91 ^{ab}	55.90±7.84 ^{de}	41.34±3.53 ^{d}	142.57±16.40 ^{cde}	144.67±15.37 ^{abc}
VM19	163.94±10.73 ^{ab}	142.77±17.33 ^{ab}	75.90±7.6 8^{a}	66.44±6.99 ^{a}	162.57±11.33 ^{abc}	162.77±12.93 ^{a}
VM22	145.62±16.9 7^{b}	121.84±10.02 ^{bcd}	70.39±7.75 ^{abc}	62.84±6.50 ^{ab}	163.72±11.97 ^{ab}	158.51±14.44 ^{a}
VM23	156.09±20.00 ^{b}	125.88±9.80 ^{bcd}	49.16±4.54 ^{e}	52.92±5.22 ^{bc}	139.16±17.59 ^{de}	125.17±11.01 ^{c}
Mean	154.58	134.79	61.78	54.59	145.84	139.95
SD	4.36	11.76	9.66	11.21	13.87	13.44
CV (%)	8.64	10.39	15.64	20.53	9.51	9.61

The different small letter superscripts within the same column represent significant differences ($p < 0.05$) (ANOVA and LSD test). The data are mean values of three replicates and standard deviation of the mean.
